# Bullying in the American Graduate Medical Education System: A National Cross-Sectional Survey

**DOI:** 10.1371/journal.pone.0150246

**Published:** 2016-03-16

**Authors:** Amar R. Chadaga, Dana Villines, Armand Krikorian

**Affiliations:** 1 Department of Internal Medicine, Advocate Health Care, Oak Lawn, Illinois, United States of America; 2 Department of Administration, Advocate Health Care, Chicago, Illinois, United States of America; 3 University of Illinois at Chicago, Department of Medicine, Chicago, Illinois, United States of America; 4 Rosalind Franklin University of Medicine and Science, Department of Medicine, North Chicago, Illinois, United States of America; Northwestern University, UNITED STATES

## Abstract

**Objectives:**

To deliver an estimate of bullying among residents and fellows in the United States graduate medical education system and to explore its prevalence within unique subgroups.

**Design/Setting/Participants:**

A national cross-sectional survey from a sample of residents and fellows who completed an online bullying survey conducted in June 2015. The survey was distributed using a chain sampling method that relied on electronic referrals from 4,055 training programs, with 1,791 residents and fellows completing the survey in its entirety. Survey respondents completed basic demographic and programmatic information plus four general bullying and 20 specific bullying behavior questions. Between-group differences were compared for demographic and programmatic stratifications.

**Main Outcomes/Measures:**

Self-reported subjected to workplace bullying from peers, attendings, nurses, ancillary staff, or patients in the past 12 months.

**Results:**

Almost half of the respondents (48%) reported being subjected to bullying although both those subjected and not subjected reported experiencing ≥ 1 bullying behaviors (95% and 39% respectively). Attendings (29%) and nurses (27%) were the most frequently identified source of bullying, followed by patients, peers, consultants and staff. Attempts to belittle and undermine work and unjustified criticism and monitoring of work were the most frequently reported bullying behaviors (44% each), followed by destructive innuendo and sarcasm (37%) and attempts to humiliate (32%). Specific bullying behaviors were more frequently reported by female, non-white, shorter than < 5’8 and BMI ≥ 25 individuals.

**Conclusions/Relevance:**

Many trainees report experiencing bullying in the United States graduate medical education programs. Including specific questions on bullying in the Accreditation Council for Graduate Medical Education annual resident/fellow survey, implementation of anti-bullying policies, and a multidisciplinary approach engaging all stakeholders may be of great value to eliminate these pervasive behaviors in the field of healthcare.

## Introduction

Over three decades have passed since Henry Silver first shined a light on the potential of abuse in medical education.[1] While mistreatment was confirmed in undergraduate medical education during ensuing years,[2] it was not until 1997 that the focus briefly turned to bullying among medical residents in the United States.[3]

In that year Baldwin and Daugherty published data involving senior students from 10 U.S. medical schools regarding their experiences with perceived mistreatment followed two years later with a matching set of questions when these students were presumably in their second year of residency.[3] Interestingly, almost every one of the 571 PGY-2s who responded experienced at least one instance of perceived mistreatment (98.6%). The types of mistreatment studied included humiliation/belittlement, others taking credit for one’s work, assigned tasks for punishment, threats to one’s career or reputation, physical abuse, sexual harassment, or disparaging comments about career in medicine.[3]

The following year, working again with Baldwin, Daugherty et al published a national cross sectional study of medical internship surveying a random 10% sample of all second-year residents listed in the American Medical Association’s database.[4] A total of 1277 surveys were returned and over 90% of residents described experiencing at least 1 incident of perceived mistreatment, with 53% reporting being belittled or humiliated by more senior residents.[4]

Bullying has been defined inclusively by Lyons as “persistent, offensive, abusive, intimidating, malicious or insulting behavior, abuse of power or unfair penal sanctions which makes the recipient feel upset, threatened, humiliated or vulnerable which undermines their self-confidence and which may cause them to suffer stress”.[5] Bullying has a significant effect on individuals in general and physicians in particular. Bullied doctors are reportedly least satisfied with their job, take more sick time, and are more likely to decrease the number of hours worked in the subsequent 12 months after being mistreated.[6] They are also more likely to cease direct patient care in the next 5 years.[6]

To our knowledge, there has been a paucity of national investigations of bullying in graduate medical education (GME) published since 1998. We hypothesized that bullying continues to be a substantial and unrecognized issue in American medical training programs especially among distinct populations. Given this premise, we aimed to deliver an updated estimate of bullying among residents and fellows in the United States, to explore the prevalence of this mistreatment associated within various and unique GME subgroups, and to provide recommendations to validate this data and enhance the GME training experience.

## Methods

The study was a cross–sectional survey conducted for 21 days in June 2015. Contact information for GME programs was obtained via the American Medical Association’s FREIDA Online®, a database with over 9,600 GME programs accredited by the Accreditation Council for Graduate Medical Education (ACGME).

An initial electronic message was sent to U.S. residency and fellowship programs asking for participation in a national bullying in GME survey. According to the ACGME, in the academic year 2013–2014, there were approximately 9,600 ACGME-accredited residency and fellowship programs constituting over 120,000 trainees.[7] The initial invitation was sent to 4,055 of these programs sampling 16 different residency specialties and 9 different internal medicine fellowships with an overall estimated 76,034 residents and fellows. In order to preserve anonymity of participants and select the most populous specialties, the 16 residency specialties each had at least one thousand residents and the 9 different internal medicine sub-specialties each had at least one hundred accredited programs. The number of residents and sub-specialty programs were confirmed via the FREIDA Online® database. This sample represented roughly 42% and 63% of the total number of programs and trainees respectively in the FREIDA Online® database.

The survey was distributed by the study authors using a chain sampling method (i.e., referral or snowball sampling) to increase reach. To participate in the study, programs were asked to forward an email invite to their respective trainees with an embedded anonymous survey link disseminated one week after the initial informative communiqué. Programs were informed they could opt out of the study by simply deleting the invitation email and were not required to notify investigators if they intended to participate or not. The study investigators did not personally contact residents or fellows nor did they have access to their personal information. A reminder email invite with the embedded survey link was sent three weeks after the initial notice.

### Ethics Statement

The Advocate Health Care Institutional Review Board reviewed and subsequently approved this study on April 21, 2015 (IRB ID 5996). The survey contained a cover page stating responses were anonymous and voluntary and would have no impact on the participants’ residency or fellowship training. By responding to the questions, the subjects agreed to participate in the research.

### Statistical Analysis

We performed an analysis of all the completed questionnaire data. Categorical variables were summarized with percentages for each group and the between group differences were assessed using chi-square tests. Survey responses were captured on a four-point scale ranging from “no” to “frequently” and were dichotomized to no and yes with yes representing “rarely”, “a few times”, and “frequently”. Analysis groups were defined as: age (≤ 30 or > 30 years old), gender, ethnicity (white or non-white), height (< 5’8” or ≥ 5’8”), body mass index (< 25 or ≥ 25), medical school (International or U.S.), resident status (Permanent Green Card/J-1/H-1B Visa or U.S. citizen) and post graduate year (PGY1 or PGY2-8). Between-group comparisons were not performed for this report regarding sexual orientation due to the disparity in group sample sizes and between the specialties and sub-specialties for economy of space. Analysis was performed using SPSS 22® (Chicago, IL) and statistical significance was determined at p < 0.05.

### Questionnaire

The questionnaire consisted of two sections (see S1 Appendix). The first collected demographic information. Ethnicity was included as minority groups may be particularly vulnerable to bullying due to discrimination and cultural differences may influence perceptions of mistreatment. The Lyons definition of bullying[5], used in previous studies, was then presented. The second part of the survey collected participant’s experience of bullying in last 12 months from peers, attending faculty, nurses, ancillary staff, or patients. Resident and fellows were asked to indicate whether they had been subjected to workplace bullying, whether they had witnessed others being bullied, who subjected them to bullying, and to what extent their health had been affected by mistreatment. Participants also completed a 20-item standardized bullying scale, asking about experience of 20 bullying behaviors in the past 12 months irrespective of whether or not they felt they had been bullied. Participants were able to navigate backwards in the survey. This scale has been validated previously in studies conducted in the United Kingdom.[8,9]

## Results

### Response Rate

Two thousand one hundred fifty-eight responses were received, with over 80% of participants (1,791) completing the questionnaire in its entirety. Twenty-eight programs (0.7%) could not be contacted due to incorrect email addresses listed in the FREIDA Online® database. Because chain sampling was used, the response rate could not be calculated.

### Demographics

Table 1 shows the participants’ age, gender, background in medicine, residency status, post graduate year (PGY), ethnicity, sexual orientation, height, body mass index (BMI), and geographic location while Table 2 shows the breakdown by specialty or sub-specialty.

**Table 1:** Demographics & Profile of Participants.

	Count (percentage)
Age	
30 and below	949 (53%)
31 and above	825 (46%)
Prefer not to say	17 (1%)
Sex	
Female	929 (52%)
Male	845 (47%)
Prefer not to say	17 (1%)
Background in Medicine	
Graduate of U.S. Medical School	1470 (82%)
Graduate of International Medical School	297 (17%)
Prefer not to say	24 (1%)
Residency Status	
United States Citizen	1609 (90%)
Permanent Green Card/J-1/H-1B Visa	168 (9%)
Prefer not to say	14 (1%)
Post Graduate Level Position	
PGY-1	389 (22%)
PGY-2 –PGY-8	1367 (76%)
Prefer not to say	35 (2%)
Race/Ethnic Group	
White	1122 (63%)
Other Ethnic Groups	541 (30%)
Prefer not to say or none of these	128 (7%)
Sexual Orientation	
Straight/Heterosexual	1653 (92%)
Other Sexual Orientation Groups	90 (5%)
Prefer not to say	50 (3%)
Height	
Under 5’8”	878 (49%)
5’8” & Above	892 (50%)
Prefer not to say	21 (1%)
Weight (BMI)	
24.9 & Below	1114 (62%)
25 & Above	651 (36%)
Prefer not to say	26 (2%)

**Table 2:** Profile of Participants by Specialty or Sub-Specialty.

	Count (percentage)
Specialty	
Anesthesiology	84 (5%)
Emergency Medicine	160 (9%)
Family Medicine	155 (9%)
Internal Medicine	195 (11%)
Neurological Surgery	27 (1%)
Neurology	53 (3%)
Obstetrics & Gynecology	126 (7%)
Ophthalmology	19 (1%)
Orthopedic Surgery	58 (3%)
Otolaryngology	44 (3%)
Pathology-Anatomic & Clinical	67 (4%)
Pediatrics	145 (8%)
Psychiatry	105 (6%)
Radiology-Diagnostic	79 (4%)
Surgery-General	96 (5%)
Urology	65 (4%)
Prefer not to say	19 (1%)
Internal Medicine Sub-Specialty	
Cardiovascular Disease	34 (2%)
Endocrinology, Diabetes, & Metabolism	33 (2%)
Gastroenterology	28 (1%)
Geriatric Medicine	11 (1%)
Hematology & Oncology	64 (4%)
Infectious Disease	28 (1%)
Nephrology	20 (1%)
Pulmonary Disease & Critical Care Medicine	61 (3%)
Rheumatology	15 (1%)

Slightly over 50% of respondents were aged thirty years old or younger. The sample was almost equally divided between men and women with most being citizens of the United States and graduating from an American medical school. International medical school graduates comprised nearly 17% of completed questionnaires while PGY-1 residents constituted 20% of the cohort. Over 60% of respondents were white and the sample was overwhelmingly straight/heterosexual in regards to reported sexual orientation. Participants were balanced in regards to height with roughly half being over or under 5’8” and almost 40% of the sample self-reported as overweight or obese. Completed surveys represented 25 different specialties and subspecialties with the highest response from Internal Medicine.

### General Bullying Experience and Between Group Differences

**Fig 1:**
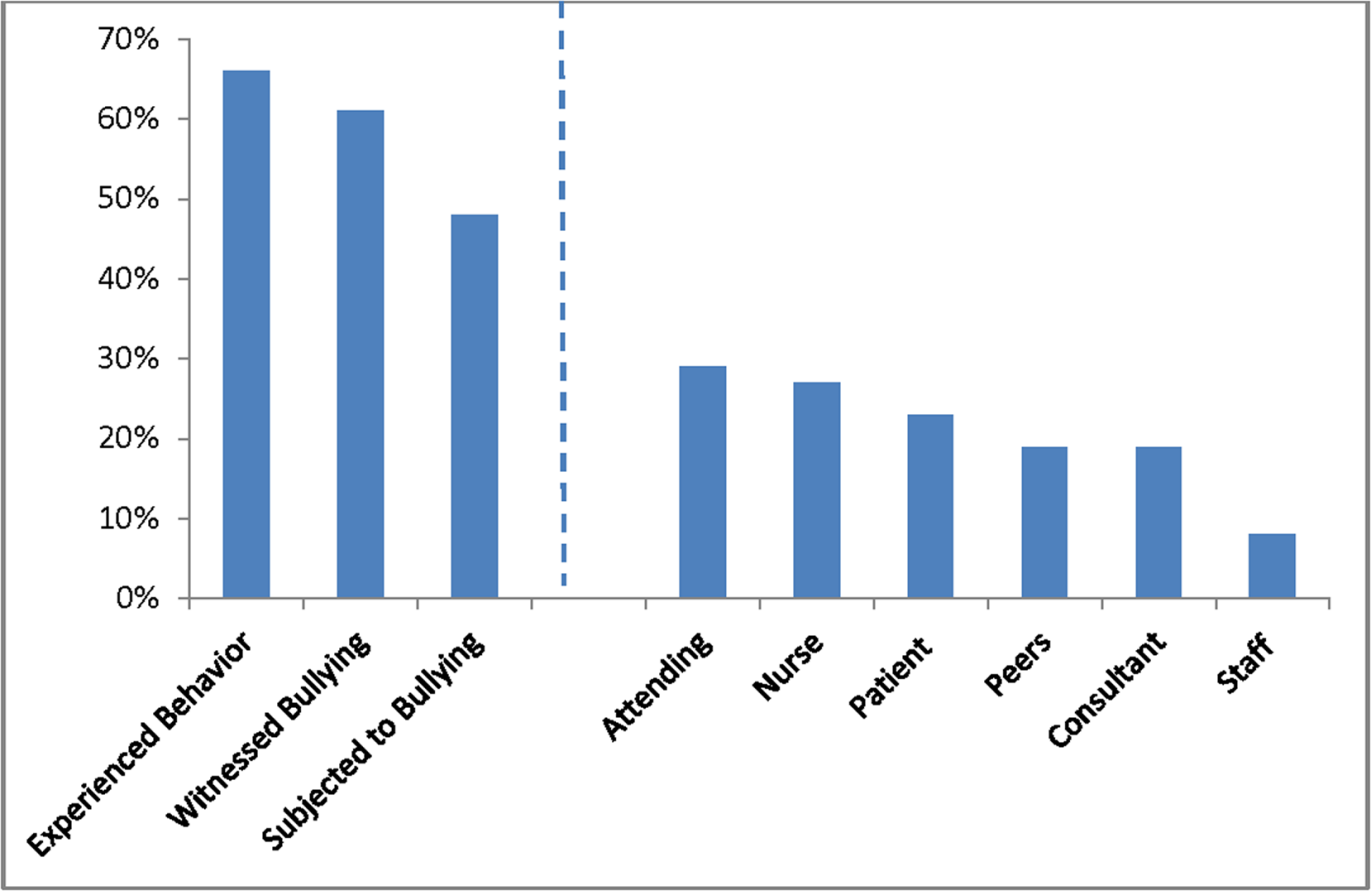
Percentage of participants who experienced bullying behaviors, witnessed bullying of colleagues, or were subjected to bullying themselves, and the source of that bullying. “Experienced behavior” was defined as a participant indicating they had experienced one or more of the specific bullying behaviors while “witnessed” and “subjected” were individual questions in the survey.

Fig 1 shows the percentage of those who experienced bullying behaviors, witnessed bullying of colleagues, were subjected to bullying themselves, and the source of those subjected to bullying. “Experienced behavior” was defined as a participant indicating they had experienced one or more of the specific bullying behaviors while “witnessed” and “subjected” were individual questions in the survey. Overall, 48% of participants reported having been bullied in the past year and 95% of these respondents stated that they had experienced one or more of the bullying behaviors. In contrast, 39% reported experiencing one or more of the bullying behaviors despite having initially indicated being part of the group (52%) who reported they had not been subjected to bullying. Sixty-one percent of all participants witnessed colleagues being subjected to workplace bullying. Ninety-seven percent of those who had been subjected to bullying themselves witnessed their colleagues being bullied. In contrast, only 29% of those who had not been subjected to bullying witnessed their peers being mistreated. Attendings and nurses were most frequently identified as the source of perceived bullying (29% and 27%, respectively) followed by patients (23%), peers (19%), consultants (19%), and ancillary staff (8%). Over a third of participants (36%) reported more than one source of bullying. Thirty-five percent of participants felt that their health had been being affected by bullying. For this subgroup, 52% reported they were subjected to bullying, 62% reported they witnessed bullying and 96% reported they had experienced bullying (data not shown).

The risk of being subjected to bullying did differ between some demographic groups with more females (52%) than males (43%, p ≤ 0.01) and more ≤ 30 years old (50%) than > 30 years old (44%, p ≤ 0.01) reporting bullying. White participants reported less bullying than non-white participants (45% versus 50%, p ≤ 0.05) and participants < 5’8” tall reported more bullying than those ≥ 5’8” (50% versus 44%, p ≤ 0.01). The risk of being subjected to bullying did not differ statistically between BMI groups, International versus U.S. medical school graduates, Permanent Green Card/J-1/H-1B Visa versus U.S. Citizen, or PGY rank.

### Specific Bullying Behaviors and Between Group Differences

In addition to the general bullying experience, participants were asked to report whether or not they had experienced 20 specific bullying behaviors (Table 3). Attempts to belittle and undermine work and unjustified criticism and monitoring of work were the most frequently endorsed behaviors (44% each) followed by destructive innuendo and sarcasm (37%) and attempts to humiliate (32%).

**Table 3:** Percentages of Bullying Behaviors Experienced by Personal Characteristics.

	Total Samplen = 1791	Gender		Ethnicity		Age		Height		BMI	
		Male n = 929	Femalen = 845	White n = 1122	Non-white n = 597	< 30 n = 949	≥ 30 n = 825	< 5’8” n = 878	≥ 5’8” n = 892	< 25 n = 1114	≥ 25 n = 651
Subjected to bullying	48	43**	52	45*	50	50**	44	50**	44	47	48
Attempts to belittle and undermine work	44	39**	49	41*	46	46*	41	47**	40	44	43
Unjustified criticism and monitoring of work	44	41*	46	40**	48	44	43	46*	41	42	46
Attempts to humiliate	32	31	34	29**	37	33	32	35*	30	31	35
Intimidating use of discipline or competence	23	24	22	20**	28	21	25	23	23	21**	26
Undermining personal integrity	26	23**	29	23**	30	24	27	27	23	25	27
Destructive innuendo and sarcasm	37	35*	40	36	39	38	37	39	36	37	38
Verbal and non-verbal threats	16	17	14	14*	17	13**	18	15	16	15	18
Inappropriate jokes	22	20**	25	18**	28	23	22	24*	20	22	23
Teasing	18	18	18	18	18	19	17	18	18	19	18
Physical violence	3	3	2	2	3	3	2	2	3	2	3
Violence to property	3	3	2	2	3	3	2	3	2	2*	3
Withholding necessary information	16	13**	19	15	17	16	17	19**	13	15*	19
Freezing out, ignoring, or excluding	25	20**	30	23*	28	25	25	28**	21	24	27
Unreasonable refusal of applications for leave, training, or promotion	9	8	9	7**	11	8	10	9	8	8*	10
Undue pressure to produce work	21	19*	23	21	21	20	23	22	19	21	21
Setting impossible deadlines	15	13	16	15	13	13	16	15	14	14	15
Undervaluing efforts	29	26**	33	29	29	29	30	31	27	28	32
Attempts to demoralize	20	20	21	19	22	18**	23	20	20	18**	23
Removal of responsibility without consultation	11	10	12	10	12	10	12	12	9	9**	13
Discrimination on racial or sexual grounds	9	7**	12	6**	14	8	10	11**	7	9	10

* represents p ≤ 0.05

** represents p ≤ 0.01

#### Personal Characteristics

Specific bullying behaviors were more frequently reported by the female, non-white, shorter than < 5’8, and BMI ≥ 25 groups. A bullying behavior profile was not consistent for age groups however despite, as noted previously, those ≤ 30 years old were bullied more than trainees who were > 30 years old. Between group differences are displayed in Table 3 and were most frequent with the gender and ethnicity groups. Females reported the following bullying behaviors more than males: attempts to belittle and undermine work (49% versus 39%, p ≤ 0.01), unjustified criticism and monitoring of work (46% versus 41%, p ≤ 0.05), undermining personal integrity (29% versus 23%, p ≤ 0.01), destructive innuendo and sarcasm (40% versus 35%, p ≤ 0.05), inappropriate jokes (25% versus 20%, p ≤ 0.01), withholding necessary information (19% versus 13%, p ≤ 0.01), freezing out, ignoring, or excluding (30% versus 20%, p ≤ 0.01), undue pressure to produce work (23% versus 19%, p ≤ 0.05), undervaluing efforts (33% versus 26%, p ≤ 0.01) and discrimination on racial or sexual grounds (12% versus 7%, p ≤ 0.01). The non-white group reported the following bullying behaviors more than the white group: attempts to belittle and undermine work (46% versus 41%, p ≤ 0.05), unjustified criticism and monitoring of work (48% versus 40%, p ≤ 0.01), attempts to humiliate (37% versus 29%, p ≤ 0.01), intimidating use of discipline or competence (28% versus 20%, p ≤ 0.01), undermining personal integrity (30% versus 23%, p ≤ 0.01), verbal and non-verbal threats (17% versus 14%, p ≤ 0.05), inappropriate jokes (28% versus 18%, p ≤ 0.01), freezing out, ignoring, or excluding (28% versus 23%, p ≤ 0.05), unreasonable refusal of applications for leave, training, or promotion (11% versus 7%, p ≤ 0.01), and discrimination on racial or sexual grounds (14% versus 6%, p ≤ 0.01).

Between group differences for the < 5’8 group in comparison to the ≥ 5’8 group were: attempts to belittle and undermine work (47% versus 40%, p ≤ 0.01), unjustified criticism and monitoring of work (46% versus 41%, p ≤ 0.05), attempts to humiliate (35% versus 30%, p ≤ 0.05), inappropriate jokes (24% versus 20%, p ≤ 0.05), withholding necessary information (19% versus 13%, p ≤ 0.01), freezing out, ignoring, or excluding (28% versus 21%, p ≤ 0.01) and discrimination on racial or sexual grounds (11% versus 7%, p ≤ 0.01). Between group differences for the BMI ≥ 25 group in comparison to the < 25 group were: intimidating use of discipline or competence (26% versus 21%, p ≤ 0.01), violence to property (3% versus 2%, p ≤ 0.05), withholding necessary information (19% versus 15%, p ≤ 0.05), unreasonable refusal of applications for leave, training, or promotion (10% versus 8%, p ≤ 0.05), attempts to demoralize (23% versus 18%, p ≤ 0.01) and removal of responsibility without consultation (13% versus 9%, p ≤ 0.01). The ≤ 30 age group reported more attempts to belittle and undermine work and unjustified criticism and monitoring of work than the > 30 age group (46% versus 41%, p ≤ 0.05). Conversely, the > 30 age group reported more verbal and non-verbal threats (18% versus 13%, p ≤ 0.05) and attempts to demoralize (23% versus 18%, p ≤ 0.01) than the ≤ 30 age group.

#### Professional Characteristics

International medical school graduates, Permanent Green Card/J-1/H-1B Visa trainees, and PGY2-8 respondents reported more specific bullying behaviors than the U.S. medical school graduates, U.S. citizens, and PGY1 participants respectively (Table 4). The International medical school group, in comparison to the U.S. group, reported more intimidating use of discipline or competence (29% versus 22%, p ≤ 0.01), verbal and non-verbal threats (22% versus 14%, p ≤ 0.01), unreasonable refusal of applications for leave, training, or promotion (13% versus 8%, p ≤ 0.01), and discrimination on racial or sexual grounds (13% versus 9%, p ≤ 0.05). The Permanent Green Card/J-1/H-1B Visa group, in comparison to the U.S. citizen group, reported more unjustified criticism and monitoring of work (51% versus 43%, p ≤ 0.05), intimidating use of discipline or competence (31% versus 22%, p ≤ 0.01), verbal and non-verbal threats (24% versus 15%, p ≤ 0.01), inappropriate jokes (30% versus 21%, p ≤ 0.01), freezing out, ignoring, or excluding (34% versus 24%, p ≤ 0.01), unreasonable refusal of applications for leave, training, or promotion (13% versus 8%, p ≤ 0.05), and discrimination on racial or sexual grounds (20% versus 8%, p ≤ 0.01). Post graduate year 2–8, in comparison to PGY1, reported more undermining personal integrity (27% versus 20%, p ≤ 0.01) and verbal and non-verbal threats (17% versus 11%, p ≤ 0.01).

**Table 4:** Percentages of Bullying Behaviors by Professional Characteristics.

	Medical School		Residence		PGY	
	IMG n = 297	US n = 1470	Visa/Green Card n = 168	U.S. Citizen n = 1609	1 n = 389	2–8 n = 1367
Subjected to bullying	46	48	45	48	50	46
Attempts to belittle and undermine work	43	44	45	44	43	44
Unjustified criticism and monitoring of work	46	43	51*	43	42	44
Attempts to humiliate	34	32	35	32	33	32
Intimidating use of discipline or competence	29**	22	31**	22	21	23
Undermining personal integrity	29	25	30	25	20**	27
Destructive innuendo and sarcasm	37	37	38	37	37	37
Verbal and non-verbal threats	22**	14	24**	15	11**	17
Inappropriate jokes	26	21	30**	21	20	23
Teasing	18	18	20	18	19	18
Physical violence	3	2	4	2	1	3
Violence to property	3	2	2	2	1	3
Withholding necessary information	20	15	19	16	16	16
Freezing out, ignoring, or excluding	28	24	34**	24	28	24
Unreasonable refusal of applications for leave, training, or promotion	13**	8	13*	8	8	9
Undue pressure to produce work	25	20	25	21	21	21
Setting impossible deadlines	16	14	17	14	15	14
Undervaluing efforts	30	29	35	29	32	28
Attempts to demoralize	22	20	24	20	17	21
Removal of responsibility without consultation	13	10	12	11	12	10
Discrimination on racial or sexual grounds	13*	9	20**	8	8	9

* represents p ≤ 0.05

** represents p ≤ 0.01

## Discussion

Our results generally confirm that bullying remains prevalent in the GME arena: forty-eight percent of residents and fellows who participated in this study reported being bullied in the previous year though 66% of trainees had in fact experienced at least one type of bullying behavior. Our findings share similarities with others found in the literature. Four years after Daugherty’s cross sectional study, Lyn Quine published results of bullying in the United Kingdom where 37% of 594 junior doctors identified themselves as having been bullied in the past year, with an overwhelming majority (84%) having actually experienced one or more bullying behaviors described on the 20 point bullying scale.[9] Black, Asian and female trainees were significantly more likely to report being bullied. In subsequent years similar rates of mistreatment amongst medical trainees have been discovered within the Irish Health System as well as the countries of India, Pakistan, Australia, and Saudi Arabia.[10–14]

Thirty-five percent of physicians in this study reported their health being affected by bullying while 61% percent had witnessed the bullying of others suggesting that mistreatment is not simply in mind’s eye of those bullied. It is interesting to note that 97% of those who experienced bullying also reported witnessing it while only 29% of participants who did not experience bullying witnessed it in others. This does raise the possibility that being subjected to bullying sensitizes one to noticing it more frequently in others.

Attendings and nurses were more frequently identified as the source of bullying (29% and 27%, respectively) followed surprisingly by patients (23%) and peers (19%). Fellows were not included as a group for the source of bullying, so they could be included as a peer or supervisor. It is important to place our results in the larger perspective. Nurses have been reported to experience bullying by physicians[15] while junior doctors have been found most likely to be bullied by their immediate team leader.[16] Nurses, residents, fellows, medical students and patients tend to occupy the base of the health care hierarchy making them prime targets for mistreatment. As each of these disenfranchised cohorts are exposed to rudeness and bullying, common bad behaviors can become contagious amongst them.[17]

Non-white trainees were significantly more likely to report being bullied than white participants. These results however could be confounded by the fact that international medical school graduates and those with non-U.S. citizen residency status tend to fit in the non-white ethnicity group. However, there were no statistically significant differences between U.S. medical school versus International white participants as well as U.S. medical school versus International non-white participants for witnessing, being subjected to or experiencing bullying (data not shown). The fact that female trainees were also more likely to be bullied raises the concern that despite a major drive towards inclusion and diversity in medical training, prevailing attitudes for a white male dominant workplace remain.

While overall bullying rates did not differ statistically between International versus U.S. medical school graduates and residency status groups, there were specific behaviors that were experienced significantly differently in these cohorts. International medical school graduates experienced significantly more intimidating use of discipline or competence, verbal and non-verbal threats, unreasonable refusal of application for leave, training or promotion, and discrimination on racial or sexual grounds than their U.S. counterparts. Similarly, those who had a Permanent green card or a J-1/H-1B Visa as opposed to being U.S. Citizens experienced significantly higher rates of inappropriate jokes and freezing out, ignoring, or excluding in addition to significantly higher rates of the behaviors International medical school graduates experienced. These results can be interpreted differently: either non-U.S. native residents experience variable degrees of non-acculturation, with subsequent misrepresentation of certain behaviors as bullying, or they are being disproportionally subjected to specific bullying behaviors. The distinction is important to pursue given that, according to the 2014 Physician Specialty Data Book, in 2013 over a quarter (25.9%) of all residents and fellows were International medical graduates.[18]

Regarding physical characteristics, our results sadly confirm the evidence rooted in pediatric literature. A report published at the turn of the century suggested that short children are more likely to be bullied than their taller peers[19] while a more recent publication states physical appearance, particularly overweight or obesity, has been reported to be a common reason for being bullied for both boys and girls.[20] While overall bullying rates did not differ statistically between those with a BMI < 25 and those with a BMI ≥ 25 in our study, those in the latter group did experience significantly more rates of intimidating use of discipline or competence, violence to property, unreasonable refusal or applications for leave, training, or promotion, attempts to demoralize, and removal of responsibility without consultation.

Our study is limited by common caveats inherent to survey designs. While we determined that 48% of residents and fellows reported being bullied in the last year, the data are cross-sectional which limits the conclusions that can be drawn about causal sequence.[16] Self-reporting of mistreatment is by definition subjective and may not correlate to the actual occurrence of bullying.[16] Although the 1791 completed questionnaires by residents and fellows constitute a larger sample than previously published reports on bullying in GME, the response rate would be low if we assumed all programs forwarded the questionnaire. In an effort to preserve anonymity, the study utilized an online survey tool to collect responses and relied on individual programs contacting their respective fellows and residents. As such, and given the opt-in and opt-out criterion, the verification of which programs forwarded the survey, if the respondents were indeed residents and fellows, and how many times they completed the study was impossible. While one could speculate that those programs with a penchant for bullying may have been disinclined to forward the study to their respective trainees, the overall prevalence in our sample was higher than some previously reported cases.[6,10,16] The higher prevalence noted in this study in turn raises the concern that those bullied were more likely to complete the questionnaire given that participation was not compulsory. This is moderately mitigated by the fact that the percentage of residents and fellows that had actually experienced at least one type of bullying behavior fits the trend of prior publications.[3,4,12,16] In addition, despite the comprehensiveness of the Lyons definition, perceptions of what constitutes bullying may vary and lead to disparate responses among participants. Finally, while the number of women and men in our survey was relatively representative of the current breakdown in GME according to the 2014 Physician Specialty Data Book[18], the number of international medical graduates and the distribution of specialties/sub-specialties was not[18], partially limiting the validity of our sample and the generalizability of this study.

Additional limitations to the interpretation of between-group differences arise from the stratification of the demographic variables. The authors selected specific cutoffs for variables such as age and height that were not evidence-based as little evidence exists to recommend the appropriate cutoffs in relation to bullying. Rather, the authors relied on their anecdotal experience as program directors to guide the appropriate cutoff and different between-group results may have been found with alternative cutoffs. Furthermore, the authors used the same height stratification for males and females despite the known height difference between the genders. While a gender specific cutoff would have been ideal, the authors could not find any published definition of the global average heights and many residents, fellows and attendings are from the international community, so it was not appropriate to select a specific countries’ averages. Finally, the authors chose to group PGY2-8 to address a specific question about whether interns differed from residents. While it is likely that many PGY5-8 are fellows, and therefore not the same as a PGY3-4, it is also possible that they are still in surgical or internal medicine advanced residencies. Therefore, the authors did not want to make assumptions about the resident/fellow categorization.

Despite these limitations, the data presented here strongly suggest that high levels of mistreatment continue to persist in today’s U.S. GME landscape. Bullying during graduate medical training appears to be part and parcel of myriad resident and fellows’ experience and perceptions. Populations unique to GME such as International medical graduates and those possessing a Permanent Green Card/J-1/H-1B Visa show a tendency to be at particularly high risk for certain bullying behaviors.

While not yet publicly available, the Association of Program Directors in Internal Medicine (APDIM) has included questions on bullying in their most recent 2015 annual program director survey. Purportedly most of the queries depend on the extrapolation of a program director’s direct knowledge of if their trainees have been bullied. Thus, the results will likely underreport the prevalence of bullying among residents given the fact that, historically, up to 90% of bullying incidents in GME went unreported.[11,12]

To our knowledge inquiries into bullying are not currently a part of the annual ACGME resident/fellow survey that monitors the climate of graduate medical clinical education and provides early warning of potential program non-compliance with ACGME accreditation standards. The inclusion of specific questions on bullying in the ACGME’s annual resident/fellow survey might mitigate the limitations of this study as well as those of the APDIM program director survey, and would validate the precise prevalence of bullying in GME across the 9,600 ACGME-accredited programs representing over 120,000 trainees. Specific institutions, specialties, sub-specialties and services will be able to obtain directed feedback about what their trainees perceive while at risk professional and personal special populations can be identified.

While the addition of questions on bullying in the ACGME annual resident/fellow survey would be a welcome first step, we believe the ACGME could strongly consider creating the tenants Lyn Quine recommended a dozen years ago: development and implementation of anti-bullying policies that include a statement on expected standards of behavior, education to raise awareness of bullying, the introduction of procedures for dealing with allegations of bullying, and protection from retaliation.[16]

The institution of these policies combined with the collection of resident and fellow data on bullying in the workplace would support the ACGME pillars of clinical learning environments characterized by excellence in clinical care, safety, and professionalism with high-quality, supervised, humanistic, clinical educational experience.[21] With these principles in place, the medical training environment could begin the long overdue transition from negative to positive and fulfill ACGME’s vision of preparing residents and fellows to become Virtuous Physicians.[21]

## Conclusion

Many trainees report experiencing bullying in the United States graduate medical education programs. Including specific questions on bullying in the Accreditation Council for Graduate Medical Education annual resident/fellow survey, implementation of anti-bullying policies, and a multidisciplinary approach engaging all stakeholders may be of great value to eliminate these pervasive behaviors in the field of healthcare.

## Supporting Information

S1 Appendix Contains questionnaire used in this study. (DOC)
