# Supplementary material for: Bullying in the American Graduate Medical Education System: A National Cross-Sectional Survey
Source: PLoS One. 2016 Mar 16;11(3):e0150246. doi: 10.1371/journal.pone.0150246 (PMC4794154; doi:10.1371/journal.pone.0150246)
Supplement: S1 Appendix — (DOC) [file pone.0150246.s001.doc]

Thank you for participating in this study of Bullying in Graduate Medical Education.

Survey responses are anonymous and voluntary and will have no impact on your training.

Data may be used for future research purposes.

1. Age:

< 20

21 – 25

26 – 30

31 – 35

36 – 40

> 41

Prefer Not to Say

1. Sex:

Female

Male

Prefer Not to Say

1. Your background in medicine:

Graduate of U.S. Allopathic Medical School (MD)

Graduate of U.S. Osteopathic Medical School (DO)

U.S. Citizen/Graduate of International Medical School

Prefer Not to Say

1. Your residency status:

United States Citizen

Permanent Resident (Green Card)

J-1/H-1B Visa Holder

Prefer Not to Say

1. Your position in the medical profession:

PGY-1  PGY-5

PGY-2  PGY-6

PGY-3  PGY-7

PGY-4  PGY-8

Prefer Not to Say

1. Your race/ethnic group:

White  Native Hawaiian/Pacific Islander

Black  American Indian/Alaska Native

Hispanic or Latino  None of these

Asian (Asian Indian, Chinese, Filipino, Japanese, Korean, Pakistani, Vietnamese, Other Asian)

Prefer Not to Say

1. Your sexual orientation (please select all that apply):

Gay  Questioning

Lesbian  Queer

Bixesual  Other

Trasngender  Not sure

Straight/heterosexual  Prefer Not to Say

1. Your Height

< 5’ 4”

5’4” – 5’ 8”

5’8” – 6’0”

> 6’0”

Prefer Not to Say

1. Your Body Mass Index (BMI)

< 18.5

18.5 – 24.9

25.0 – 29.9

30 and above

Prefer Not to Say

1. A rough estimate of where your program is located is:

Northeast

Midwest

South

West

Prefer Not to Say

1. Specialty:

Anesthesiology  Radiology-Diagnostic

Emergency Medicine  Surgery-General

Family Medicine  Urology

Internal Medicine  Cardiovascular Disease

Neurological Surgery  Endocrinology, Diabetes, & Metabolism

Neurology  Gastroenterology

Obstetrics & Gynecology  Geriatric Medicine

Ophthalmology  Hematology & Oncology

Orthopaedic Surgery  Infectious Disease

Otolaryngology  Nephrology

Pathology-Anatomic & Clinical  Pulmonary Disease & Critical Care Medicine

Pediatrics  Rheumatology

Psychiatry  Prefer Not to Say

# Workplace Bullying

In this section we would like to ask some questions about harassment and bullying at work. Workplace bullying constitutes ‘persistent, offensive, abusive, intimidating, malicious or insulting behaviour, abuse of power or unfair penal sanctions, which makes the recipient feel upset, threatened, humiliated or vulnerable, which undermines their self-confidence and which may cause them to suffer stress’.

1. In the past 12 months, have you witnessed work colleagues being subjected to workplace bullying from peers, attendings, nurses, patients, or ancillary staff?

No  Rarely  A few times  Frequently

1. In the past 12 months, have you been subjected to workplace bullying from peers, attendings, nurses, patients or ancillary staff?

No  Rarely  A few times  Frequently

1. If you have experienced bullying, please states who has subjected you to bullying.

Please state who has subjected you to bullying:

|  | Yes | No |
| --- | --- | --- |
| Peer – Intern/Resident | ◦ | ◦ |
| Supervisor – Attending | ◦ | ◦ |
| Supervisor – Consultant | ◦ | ◦ |
| Nurse | ◦ | ◦ |
| Ancillary staff – Pharmacist/Respiratory Therapist/etc. | ◦ | ◦ |
| Patients | ◦ | ◦ |

1. To what extent would you say your health has been affected by bullying?

Not at all A great deal

◦1 ◦2 ◦3 ◦4 ◦5

Please answer the following question even if you do not consider that you have been bullied.

1. In the last 12 months have you experienced from peers, attendings, consultants, nurses, patients, or ancillary staff any of the following in the workplace:

|  | No | Rarely | A few times | Frequently |
| --- | --- | --- | --- | --- |
| 1. Persistent attempts to belittle and undermine your work? | ◦ | ◦ | ◦ | ◦ |
| 1. Persistent and unjustified criticism and monitoring of your work? | ◦ | ◦ | ◦ | ◦ |
| 1. Persistent attempts to humiliate you in front of colleagues? | ◦ | ◦ | ◦ | ◦ |
| 1. Intimidating use of discipline or competence procedures? | ◦ | ◦ | ◦ | ◦ |
| 1. Undermining your personal integrity? | ◦ | ◦ | ◦ | ◦ |
| 1. Destructive innuendo and sarcasm? | ◦ | ◦ | ◦ | ◦ |
| 1. Verbal and non-verbal threats? | ◦ | ◦ | ◦ | ◦ |
| 1. Making inappropriate jokes about you? | ◦ | ◦ | ◦ | ◦ |
| 1. Persistent teasing? | ◦ | ◦ | ◦ | ◦ |
| 1. Physical violence? | ◦ | ◦ | ◦ | ◦ |
| 1. Violence to property? | ◦ | ◦ | ◦ | ◦ |
| 1. Withholding necessary information from you? | ◦ | ◦ | ◦ | ◦ |
| 1. Freezing out, ignoring, or excluding? | ◦ | ◦ | ◦ | ◦ |
| 1. Unreasonable refusal of applications for leave, training, or promotion? | ◦ | ◦ | ◦ | ◦ |
| 1. Undue pressure to produce work? | ◦ | ◦ | ◦ | ◦ |
| 1. Setting of impossible deadlines? | ◦ | ◦ | ◦ | ◦ |
| 1. Constant undervaluing of your efforts? | ◦ | ◦ | ◦ | ◦ |
| 1. Persistent attempts to demoralise you? | ◦ | ◦ | ◦ | ◦ |
| 1. Removal of areas of responsibility without consultation? | ◦ | ◦ | ◦ | ◦ |
| 1. Discrimination on racial or sexual grounds? | ◦ | ◦ | ◦ | ◦ |
